# Announcement: Symposium on “Metal Ions in Biology and Medicine: Natural and Synthetic Approaches”

**DOI:** 10.1155/MBD.1995.189

**Published:** 1995


					SYMPOSIUM ON "METAL IONS IN BIOLOGY AND MEDICINE:
NATURAL AND SYNTHETIC APPROACHES"
to be held at the
1995 International Chemical Congress of Pacific Basin Societies
December 17 22, 1995, Honolulu, Hawaii
Organizers
Ann English, Department of Chemistry and Biochemistry, Concordia University, Montreal, H3G
1M8, Canada; Phone: 514-848-3338; Fax: 514-848-2868; Email: English@vax2.concordia.ca
Ichiro Okura, Department of Bioengineering, Tokyo Institute of Technology, Yokohama, Japan;
Phone: 045-924-5752; Fax: 045-924-5778
Shigenobu Yano, Department of Chemistry, Nara Women's University, Nara, Japan; Phone/Fax:
0742-20-3392; Email: Yano@cc.nara-wu.ac.jp
Alison Butler, Department of Chemistry, University of California at Santa Barbara, Santa 9arbara,
CA, 93106, USA; Phone: 805-893-8178; Fax: 805-893-4120; Email: Butler@sbmml.ucsb.edu
John Dawson, Department of Chemistry & Biochemistry, University of South Carolina, Columbia,
SC, 29208, USA; Phone: 803-777-7234; Fax: 803-777-9521; Email: Dawson@chem.scarolina.edu
Session 1. Hydrocarbon Oxygenation by Metalloenzymes and Synthetic Models
Chairs: John Dawson and Ann English
John Groves, Princeton University, USA, "Models for Oxygen Activation in Membranes"
David Dolphin, University of British Columbia, Canada, "Models for Heine Enzymes"
Yoshihito Watanabe, Institute of Molecular Science, Japan, "Roles of Proximal Ligands in Heine
Enzymes"
John Dawson, University of South Carolina, USA, "Oxygen Activation by Heine Enzymes"
COFFEE BREAK
Kenneth Karlin, Johns Hopkins University, USA, "Copper-Dioxygen Reversible Binding and
Reactivity"
Steve Sligar, University of Illinois, USA, "Hydrocarbon Oxidation and Dehalogenation Reactions
Catalyzed by Cytochrome P-450"
Stephen Lippard, Massachusetts Institute of Technology, USA, "Methane Mono-Oxygenase:
Structure and Function"
.Ann English, Concordia University, Canada, "Oxyferryl Heme Centers in Proteins"
Session 2. Peroxidases, Oxidases and Related Systems
Chairs: Alison Butler and Osamu Yamauchi
Alison Butler, University of California Santa Barbara, USA, "Mechanistic Studies of Vanadium-
Containing Peroxidases"
Jilly Evans, Merck Frosst, Canada, "Mechanistic Studies of Prostaglandin H Synthase I1"
Bruce Hill, Queen's University, "Mechanistic Studies of Cytochrome Oxidase"
Julie Kovacs, University of Washington, USA, "Spectroscopic Models for the Low-Spin, Non-
Heine, Thiolate-Ligated Fe Site in Nitril Hydratase"
COFFEE BREAK
Osamu Yamauchi, Nagoya University, Japan, "Functionalization of Aromatic Amino Acids.
Stacking, Metal Binding and Radical Formation"
Etsuo Niki, Tokyo University, Japan, "Transition Metal Catalyzed Free Radical Generation and
Oxidative Damage of Biological Molecules"
Tetsuo Nagano, Tokyo University, Japan, "Development of Novel Superoxide Dismutase Mimics in
vivo"
Vincent Pecoraro, University of Michigan, USA, "Models for Manganese-Containing Redox
Enzymes"
Session 3. New Frontiers in Bio-lnorganic Chemistry
Chairs: Shigenobu Yano and Jik Chin
Shigenobu Yano, Nara Women's University, Japan, "Bioactive Sugar Complexes"
Hiromu Sakurai, Kyoto Pharmaceutical University, Japan, "Insulin- Mimetic Antidiabetic Vanadyl
Complexes"
189
Judith Burstyn, University of Wisconsin, USA, "Soluble Guanylyl Cyclase: Structure of the Heme
Center and Mechanism"
Des Richardson, McGill University, Canada, "Iron Uptake by Cells"
COFFEE BREAK
Charles Young, University of Melbourne, Australia, "Models for Molybdenum-Containing Enzymes"
Yuki Fujii, Ibaragi University, Japan, "Models for Zinc-Containing Enzymes"
Jik Chin, McGill University, Canada, "Artificial Hydrolytic Metalloenzymes"
Toshihiko Ozawa, National Institute for Radiological Science, Japan, "Reactions of Some Cu(ll)-
Oligopeptide Complexes with Active Oxygen Species ESR Spin Trapping Studies"
Session 4. Metals in Medicine
Chairs: Ichiro Okura and Thomas Tullius
Thomas Tullius, Johns Hopkins University, USA, "Metal Complexes as Probes of DNA Structure"
Janet Morrow, State University of New York, USA, "Artificial Nucleases for Antisense
Oligonucleotide Applications"
Ichiro Okura, Tokyo Institute of Technology, Japan, "Development of Photosensitizers for
Photodynamic Therapy and Fluorescent Compounds in Tumor Cells"
Chris Orvig, University of British Columbia, Canada, "Metal Ligands for Therapeutic Uses"
Thomas O'Halloran, Northwestern University, USA, "Receptor-Targeted Drug Delivery:
Characterization of Cisplatin-Based Protein-DNA Crosslinkers"
Session 5. Kitajima Memorial Session A
Chairs: Kenneth Karlin and Lawrence Que, Jr.
Yoshihito Moro-Oka, Tokyo Institute of Technology, Japan, "Why Copper, Why Iron in the Active
Centers of Mono-Oxygenases"
Edward Solomon, Stanford University, USA, "Multicopper Oxidases and Oxygenases:
Structure/Function Correlations"
Thomas Spiro, Princeton University, USA, "Structure and Bonding in "Blue" Copper Proteins from
Resonance Raman Spectroscopy and Model Compound Studies"
Kazuyuki Tatsumi, Nagoya University, Japan, "Theoretical Aspects of Kitajima's Dioxygen
Complexes"
Lawrence Que, Jr., University of Minnesota, USA, "Models for Non-Heine Iron Mono-Oxygenases"
Session 6. Kitajima Memorial Session B
Chairs: Yoshihiko Moro-Oka and Yoshihito Watanabe
Eiichi Kimura, Hiroshima University, Japan, "Intrinsic Properties of Zinc(ll) in Hydrolytic Enzymes"
Christopher Reed, University of Southern California, USA, "Unusual u-Hydroxy Diiron(lll)
Porphyrins: Essentially Linear and Magnetically Uncoupled Fe-(OH)-Fe Linkages"
Isao Morishima, Kyoto University, Japan, "Structural Determinants of High-Valent Heine
Formation as Studied with Model Porphyrin Complexes and Recombinant Mutant Heine Enzymes"
Teizo Kitagawa, Institute for Molecular Science, Japan, "Time-Resolved Resonance Raman Study
of the Mechanism of Dioxygen Reduction at the Binuclear Center of Cytochrome c Oxidase"
Richard Holm, Harvard University, USA, "Cuboidal Fe3S4 Clusters"
Poster Session
There will be a poster session for contributed presentations. The deadline for receipt of abstracts is
May 1, 1995. Special Pacifichem Abstract forms can be obtained from the Pacifichem '95
Secretariat which is also the address for abstract submission.
Authors of Contributed papers must also submit two copies of a 500 1000 word "long abstract"
including relevant tables, charts and figures. There is no special form for the long abstract.
Pacifichem'95 Secretariat, American Chemical Society 1155 Sixteenth Street, NW, Washington,
DC 20036 USA Phone: 202-872-4396; Fax: 202-872-6128
Please also send copies of the short and long abstracts to John Dawson at the South Carolina
address listed above.
Please note: The Congress Dates are December 17 22, 1995.
Exact dates for the Symposium have not been set at this time.
190

				

